# Resistance selection of triflumezopyrim in *Laodelphax striatellus* (fallén): Resistance risk, cross-resistance and metabolic mechanism

**DOI:** 10.3389/fphys.2022.1048208

**Published:** 2022-11-29

**Authors:** Shengfang Wen, Chang Liu, Xueting Wang, Youwei Wang, Chao Liu, Jinhua Wang, Xiaoming Xia

**Affiliations:** ^1^ College of Plant Protection, Shandong Agricultural University, Taian, China; ^2^ College of Resources and Environment, Shandong Agricultural University, Taian, China

**Keywords:** *Laodelphax striatellus*, triflumezopyrim, resistance risk, biological fitness, detoxification enzyme

## Abstract

The risk assessment and resistance mechanisms of insecticide resistance are critical for resistance management strategy before a new insecticide is widely used. Triflumezopyrim (TFM) is the first commercialized mesoionic insecticide, which can inhibit nicotinic acetylcholine receptor with high-performance against the small brown planthopper (SBPH), *Laodelphax striatellus* (Fallén). In our study, the resistance of SBPH to TFM increased 26.29-fold, and the actual heritability of resistance was 0.09 after 21 generations of continuous selection by TFM. After five generations of constant feeding under insecticide-free conditions from F_16_ generation, the resistance level decreased 2.05-fold, and the average resistance decline rate per generation was 0.01, but there were no statistical decline. The TFM resistant strains had no cross-resistance to imidacloprid, nitenpyram, thiamethoxam, dinotefuran, flonicamid, pymetrozine, and chlorfenapyr. The third and fifth nymphal stage duration, pre-adult stage, adult preoviposition period, longevity, emergence rate, and hatchability of the resistant strain were significantly lower than those of the susceptible strain, while the female-male ratio was considerably increased. The fitness cost was 0.89. Further, cytochrome P450 monooxygenase (P450) and carboxylesterase (CarE) activities were markedly increased, but only the enzyme inhibitor piperonyl butoxide (PBO) had a significant synergistic effect on the resistant strain. The expression of *CYP303A1*, *CYP4CE2,* and *CYP419A1v2* of P450 genes was significantly increased. SBPH has a certain risk of resistance to TFM with continuous application. The TFM resistance may be due to the increased activity of P450 enzyme regulated by the overexpression of P450 genes.

## 1 Introduction

The small brown planthopper (SBPH), *Laodelphax striatellus* (Fallén), is one of the most economically important pests widely distributed in China (Zheng et al., 2017). SBPH can damage rice, corn, and wheat by sucking plants, ovipositing, and spreading viral diseases, leading to severe crop yield reduction (Mu et al., 2016; Wei et al., 2017; Wu et al., 2018). Currently, insecticides are the primary measure to control SBPH in the field (Miah et al., 2018). However, SBPH has developed varying degrees of resistance to several insecticides, including chlorpyrifos, ethiprole, buprofezin, and imidacloprid, as a result of their long-term, extensive, and unreasonable application (Gao et al., 2008; Wang et al., 2008; Xu et al., 2014; Elzaki et al., 2015; Liu et al., 2015).

Triflumezopyrim (TFM) is a new mesoionic insecticide that acts on nicotinic acetylcholine receptors (nAChRs) (Cordova et al., 2016; Wen et al., 2021a). TFM is the only available acetylcholine receptor inhibitor (Cordova et al., 2016). TFM has a wide insecticidal spectrum with a long-lasting effect. Further, it exerts an excellent insecticidal effect on rice planthoppers (Zhang K. L et al., 2020; Wen et al., 2021b). TFM has great potential in the integrated control and resistance management of SBPH. Previous researches reported that sublethal doses of TFM could significantly affect population development and detoxification enzyme activities of *Sogatella furcifera* (Horvath) (Chen et al., 2020) and SBPH (Wen et al., 2021b). The new study reported that SBPH had a certain risk of resistance to TFM after a short-term continuous selection (Zhang et al., 2022).

The risk assessment of insecticide resistance is crucial before continuous application. This will provide in-depth knowledge about applying new insecticides to control a specific pest comprehensively. It can be assessed by the realistic heritability (*h* ^*2*^), fitness cost, resistance stability, and cross-resistance (Wang et al., 2021). The *h* ^*2*^ can estimate the genetic ability and risk of resistance (Afzal and Shad, 2016). Fitness cost refers to the disadvantage of the fitness of resistance genes, manifested in the ecological, physiological, or biochemical changes of individual organisms and populations to adapt to the adverse effects of pesticides, such as decreased fecundity and survival rate. It may affect the development of insecticide resistance (Kliot and Ghanim, 2012; Tieu et al., 2017). Several studies have reported the fitness cost of planthopper against imidacloprid, chlorfluazuron, buprofezin, etc (Liu and Han, 2006; Liao et al., 2019b; Zeng et al., 2022). Resistance stability can predict the development trend of resistance. The more stable the resistance is, the more difficult it is to manage (Afzal et al., 2015). The determination of cross-resistance can provide ideas for alternation of pesticides and the resistance mechanism (Wang R. et al., 2020).

Therefore, we screened SBPH strain resistant to TFM after long-term continuous selection, evaluated the resistance risk of SBPH to TFM, and determined the cross-resistance with other insecticides. Further, the metabolic mechanism of resistance SBPH for TFM was preliminarily studied. This research provides a theoretical reference for resistance mechanism study and SBPH resistance management for TFM.

## 2 Materials and methods

### 2.1 Insects

The susceptible strain (SS) of SBPH was obtained from Yutai, Shandong Province (N35° 1′ 13.00″ E116° 38′ 3.01″) in October 2016. The SS strain was reared on ‘Wuyujing 3’ rice seedlings without exposure to insecticides for more than 2 years. The rice-seedling spraying approach was used to continuously select the resistant strain (RS) of SBPH from the SS to 21 generations. The insects were raised at 25 ± 1°C, 70%–80% relative humidity, and a 16:8 h L:D photoperiod.

### 2.2 Insecticides and synergists

TFM (96%) and cyantraniliprole (94%) were supplied by DuPont Company (Shanghai, China). Imidacloprid (96%) was provided by Shandong Weifang Rainbow Chemical Co., Ltd (Weifang, China). Dinotefuran (99.1%), thiamethoxam (98%), flonicamid (98.5%), pymetrozine (98%), and nitenpyram (98%) were purchased from Shandong United Pesticide Industry Co., Ltd (Jinan, China). Piperonyl butoxide (PBO, 95%), triphenyl phosphate (TPP, 98%), and diethyl maleate (DEM, 96%) were purchased from Shanghai Macklin Biochemical Co., Ltd (Shanghai, China).

### 2.3 Bioassay

The rice-seedling dip method described by Xu et al. (2019) and Liao et al. (2019b), with minor changes, was used to test TFM toxicity for SBPH. TFM was dissolved in acetone and diluted to different concentrations with deionized water containing 0.1% Triton X-100. Deionized water containing 0.1% Triton X-100 was employed as a control. Rice seedlings with roots were cut to a length of approximately 10 cm, rinsed with water, and air-dried in a shady environment. The rice seedlings were soaked in insecticide for 30 s, retrieved and drained until the liquid stopped dripping, and then dried naturally in the shade. Wet absorbent cotton was used to cover the roots of the rice seedlings, which were then put in glass test tubes (2 cm × 20 cm) with five seedlings per tube. The third instar nymph of SBPH was sedated with CO_2_ (10–15 s) before being put in a test tube containing rice seedlings, with 20 insects in each tube and three replicates for each concentration. Afterwards, the glass test tubes were sealed with four layers of gauze (200-mesh). The feeding conditions of all treatments were the same as those in Section 2.1, and the mortality was recorded after 72 h. If the insects did not move after being lightly touched with a small writing brush, they were pronounced dead.

### 2.4 Resistance selection

SBPH resistance to TFM was selected with minor changes of Mao et al.’s (2016) rice-seedling spraying approach. The LC_50_ value measured by the previous generation bioassay was used as the concentration of TFM in the spray to screen the resistance of the next generation. Rice seeds were placed on two moistened layer paper towels in plastic boxes (35 cm × 25 cm × 15 cm). When the rice seedlings were approximately 10 cm tall, the sufficient TFM solution prepared following above methods was uniformly sprayed onto rice seedlings by using Matabi sprayer (style 7) until the dripping water, which were then dried in a cool and dry place. Then more than 1,000 third instar nymphs were moved into the boxes containing treated rice seedlings with insecticide, and covered with a mesh cloth for feeding at the above condition. More than 4,000 third instar nymphs were treated in each screening generation. After 72 h, the surviving insects were moved into a new box containing fresh rice seedlings without insecticide for continuous routine feeding. The toxicity of TFM to SBPH was measured by rice-seedling dip method described above in each generation to monitor the development of resistance.

$$Resistance\;ratio\;\left(RR\right)=\frac{{LC}_{50}\;of\;TFM\;to\;RS}{{LC}_{50}\;of\;TFM\;to\;SS}$$

### 2.5 Estimation of the resistance realistic heritability and prediction of the resistance development rate

Tabashnik and Mcganghey (1994) and Tabashnik (1992) provided approaches for determining the *h* ^*2*^ and resistance development rates of TFM resistant strain, respectively. The specific calculation method is in Supplementary Figure S1.

### 2.6 Resistance stability

A portion of the RS-F_16_ strain was isolated individually and reared continuously for five generations without exposure to any pesticide to evaluate TFM resistance stability in SBPH, similar to the approach described by Tabashnik and Mcganghey (1994). *R* was calculated to determine the average selection response:

$$R=\frac{lg\left(finale\;{LC}_{50}\right)-lg\left(initial{\;LC}_{50}\right)}{N}$$

where N is the response of the number of generations reared without being exposed to pesticides.

### 2.7 Cross-resistance

The sensitivity of the third instar nymphs of SS and RS (F_20_) strains to neonicotinoids insecticide (imidacloprid, dinotefuran, thiamethoxam, flonicamid, nitenpyram), pyridine azomethine insecticide (pymetrozine) and anthranilic diamide insecticide (cyantraniliprole) were determined using the rice-seedling dip method described in Section 2.3 to clarify the cross-resistance of TFM with these insecticides. The following formula was used to compute the cross-resistance ratio (CR):

$$CR=\frac{{LC}_{50}\;of\;test\;insecticide\;to\;RS\;}{{LC}_{50}\;of\;test\;insecticide\;to\;SS}$$

### 2.8 Biological fitness

The biological fitness of the SS and RS strains of SBPH was evaluated using a modified version of the age-stage, two-sex life table technique reported by Liao et al. (2019a). Fifty nymphs (fifth instar) were randomly selected from the SS and RS (F_20_) strains, fed separately in glass test tubes. When the adults emerged, single female and male were paired and fed separately with fresh rice seedlings, which were inspected every half-day (8:00 and 20:00) to check whether there were nymphs on the seedlings. One hundred newly hatched nymphs were collected from the SS and RS strains on the same day. Each of the 100 nymphs was considered a replicate for each strain and individually fed in separate test tubes. The nymph’s molting and death were examined and recorded twice daily (8:00 and 20:00). The withered rice seedlings were replaced promptly to insure sufficient nutrition. The adults were separately paired when they emerged. The rice seedlings were superseded and retained every half-day (8:00 and 20:00) until all the insects died. Based on the method by Shao et al. (2012), the quantity of newly hatched nymphs was observed and documented until no nymphs hatched for 10 days. All the rice seedlings were boiled for 10 min and 48-h submerged in 95% ethanol. Then the rice seedlings were dissected, and the unhatched eggs were documented under the anatomical microscope.

### 2.9 Synergism of the enzyme inhibitors

The approach for determining the synergism of the enzyme inhibitors is similar to that described by Liao et al. (2019a), with minor variations. The enzyme synergist triphenyl phosphate (TPP), diethyl maleate (DEM), and piperonyl butoxide (PBO) were dissolved in acetone and prepared in different concentrations. The fifth instar nymphs from the RS (F_17_) and SS strains were selected as test insects. There were 20 insects in each treatment, and the experiment was repeated thrice for each concentration. SBPH was anaesthetized with CO_2_ for 20 s, and 0.04 μL of a synergist was applied to the insects pronotum by a micropipette. Acetone treatment was used as a control. Preliminary tests were undertaken to establish the maximal dose (TPP 0.2 µg/insect; DEM 2.5 µg/insect; PBO 0.2 µg/insect) of the synergists that had no apparent adverse impact on the fifth instar nymphs of SBPH for 72 h. After pretreatment with the synergist for 1 h, the effect of the synergists on the efficacy of TFM was determined by the rice-seedling dip method described in Section 2.3. The following formula was used to compute the synergistic ratio (SR):

$$SR=\frac{{LC}_{50}\;of\;TFM\;alone}{{LC}_{50}\;of\;TFM\;with\;the\;synergist}$$

### 2.10 Assays for the activity of detoxification enzymes

The extraction method of enzyme solution was according to the conventional method of Ding et al. (2021). Three replicates were used for each strain, and each replicates contained thirty third instar nymphs (90 insects per strain).

The activities of carboxylesterase (CarE), glutathione-*S*-transferase (GST), and cytochrome P450 monooxygenase (P450) were determined according to conventional methods. CarE activity was evaluated according to the methods reported by Han et al. (1998) and Ding et al. (2021). Activity determination of GST and P450 refers to the practices of Kao et al. (1989) and Aitio (1978), respectively. According to Wen et al. (2021b), the protein concentrations were measured using the Enhanced BCA (Bicinchoninic acid) Protein Assay Kit (Beyotime Biotechnology). The specific operation steps are in Supplementary Figure S2.

### 2.11 Determination of the expression levels of P450 genes

The third instar nymphs of the SS and RS (F_20_) strains were collected, and each strain was divided into three groups with 30 insects in each group. The total RNA was extracted using the RNA-esay™ Isolation Reagent (Vazyme Biotech Co., Ltd, Nanjing, China). After that, reverse transcription of RNA to first-strand cDNA was performed using HiScript^®^III RT SuperMix for qPCR (+gDNA wiper) reagent kit (Vazyme Biotech Co., Ltd, Nanjing, China). The qRT-PCR reaction was carried out using ChamQ universal SYBR^®^ qPCR Master Mix reagent kit (Vazyme Biotech Co., Ltd, Nanjing, China). The primers used in qRT-PCR analyses were provided in Supplementary Table S1, and the level of P450 gene transcripts was normalized to that of GAPDH. Relative quantification was performed using the 2^−ΔΔCT^method (Livak and Schmittgen, 2021).

### 2.12 Data analysis

SPSS 16.0 was used to calculate the LC_50_ value, slope, 95% confidence interval (CI), and χ^2^ and performed to analyze the differences in emergence rate, female-male ratio, hatchability, metabolic enzyme activities, and expression levels of P450 genes in SS and RS strains by one-way ANOVA with Tukey’s test (*p* < 0.05). The toxicity differences in different treatments for resistance stability and synergism test were compared by Poloplus software. The difference of toxicity is significant if the 95% confidence limit for the median lethal dose ratio is greater than 1 and the *p* value for the equality hypothesis is less than 0.05. TWOSEX-MSChart software was performed on analyzed SBPH life table data, including the intrinsic rate of increase (*r*), finite rate of increase (*λ*), net reproductive rate (*R*_*0*_), and mean generation time (*T*) (Chi 1988; Chi, 2020) and to compare the differences in developmental duration, single female egg production, life span, and other life table parameters of SBPH in SS and RS strains at different stages by bootstrapping (100,000 times) (Huang and Chi, 2013; Akca et al., 2015). The formulas are as follows.

$$R_{0}=\overset{\infty }{\underset{x=0}{\sum }}l_{x}m_{x}$$

$$\overset{\infty }{\underset{x=0}{\sum }}e^{-r\left(x+1\right)}l_{x}m_{x}=1$$

$$\lambda =e^{r}$$

$$T=\frac{\ln R_{0}}{r}$$

$$Relative\;fitness\;\left(R_{f}\right)=\frac{\;R_{0}\;\left(RS\right)}{R_{0}\;\left(SS\right)}$$

## 3 Results

### 3.1 Triflumezopyrim selection pressure on small brown planthopper

**FIGURE 1:**
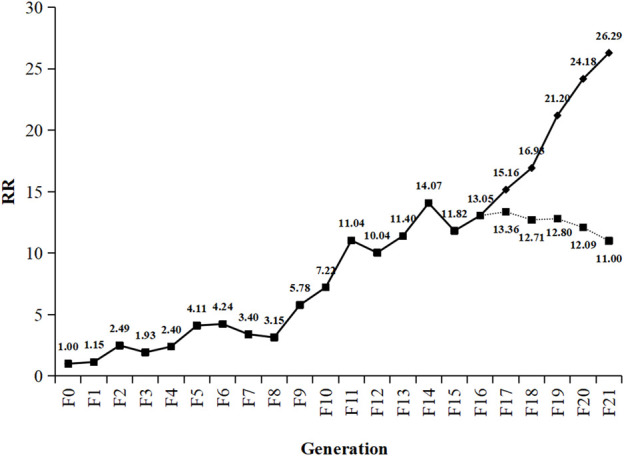
Change trend of triflumezopyrim resistance ratio from F0 to F21. Generations of *Laodelphax striatellus*. The dotted line represents the trend in resistance stability of RS strain (F16) of SBPH after continuously reared 5 generations without TFM. RR (resistance ratio) = $\frac{{LC}_{50}\;of\;TFM\;to\;RS}{{LC}_{50}\;of\;TFM\;to\;SS}$ .

The RS strain was derived from the SS strain of SBPH by continuous TFM selection of 21 generations in the laboratory (Table 1; Figure 1). The LC_50_ value of TFM for SBPH increased from 0.55 mg L^−1^–14.46 mg L^−1^. The resistance slowly increased after selection from F_0_ to F_8_ generations. The resistance ratio increased only by approximately 4-fold. However, from F_8_ to F_21_ generations, the resistance development rapidly increased, the resistance ratio of the RS strain was increased 26.29-fold. Base on the resistance levels criterion: low (RR = 5–10-fold), medium (RR = 10–100-fold), and high (>100-fold) (Mu et al., 2016; Xue et al., 2022), the resistance level was intermediate.

**TABLE 1:** The resistance development of the susceptible strain of SBPH to TFM.

Selected generations	No.	Slope±SE	LC_50_ (mg·L^−1^)	95% CI^a^	*χ* ^2^ (*df*)	Resistance ratio (RR)
F_0_	360	1.40 ± 0.27	0.55	0.39–0.88	3.64 (5)	-
F_1_	360	1.38 ± 0.27	0.63	0.44–1.07	2.10 (5)	1.15
F_2_	360	1.71 ± 0.29	1.37	0.93–1.86	2.46 (5)	2.49
F_3_	360	0.86 ± 0.26	1.06	0.58–5.50	1.28 (5)	1.93
F_4_	360	0.89 ± 0.26	1.32	0.70–8.20	2.63 (5)	2.40
F_5_	360	1.36 ± 0.27	2.23	1.55–3.61	2.95 (5)	4.05
F_6_	360	1.46 ± 0.38	2.33	1.54–5.89	2.33 (4)	4.24
F_7_	360	1.72 ± 0.35	1.87	1.25–2.49	4.17 (5)	3.40
F_8_	360	1.62 ± 0.35	1.80	1.15–2.43	5.19 (5)	3.27
F_9_	360	1.10 ± 0.42	3.18	1.77–9.84	1.20 (5)	5.78
F_10_	360	1.53 ± 0.44	3.91	2.65–8.74	3.64 (5)	7.11
F_11_	360	1.11 ± 0.25	6.02	3.53–10.56	3.10 (6)	10.95
F_12_	360	1.29 ± 0.21	5.52	3.80–7.99	7.83 (6)	10.40
F_13_	360	1.39 ± 0.33	6.40	4.10–11.45	0.89 (5)	11.64
F_14_	360	1.47 ± 0.22	7.74	5.63–11.29	4.18 (6)	14.07
F_15_	360	1.44 ± 0.34	6.50	4.25–12.91	0.66 (5)	11.82
F_16_	360	1.26 ± 0.32	7.18	4.31–11.32	2.88 (5)	13.05
F_17_	360	1.20 ± 0.26	8.34	5.38–13.17	3.77 (5)	15.16
F_18_	360	1.63 ± 0.23	9.31	7.23–12.31	7.13 (5)	16.93
F_19_	360	1.07 ± 0.26	11.66	6.17–18.37	4.57 (5)	21.20
F_20_	360	1.01 ± 0.31	13.30	5.67–25.21	2.49 (5)	24.18
F_21_	360	1.37 ± 0.27	14.46	9.63–20.97	2.44 (5)	26.29

^a^ 95% CI = 95% confidence interval.

### 3.2 Estimation of the realistic heritability of resistance and prediction of the resistance development rate

**FIGURE 2:**
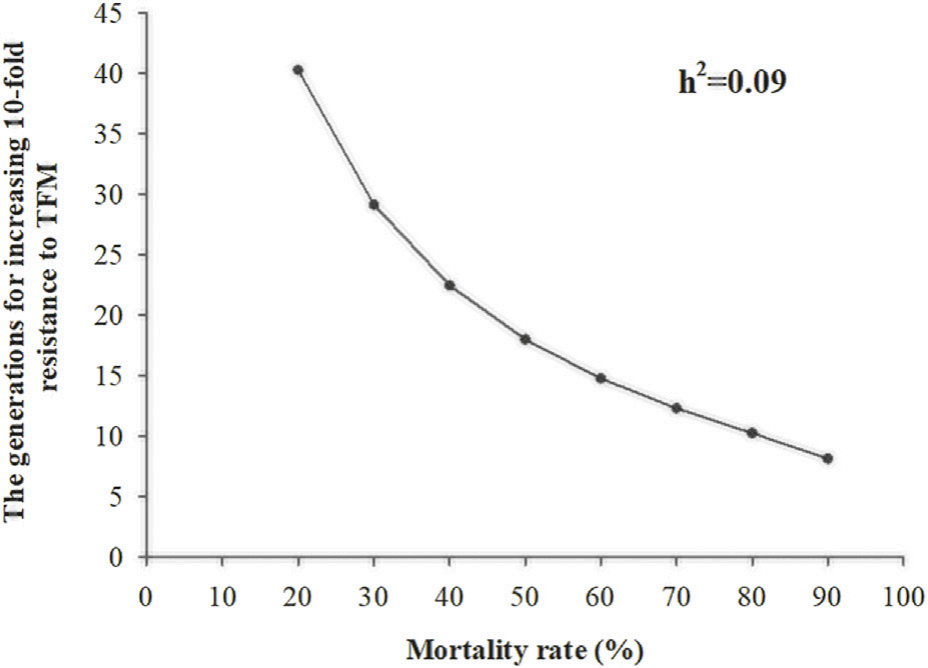
The number of generations of *Laodelphax striatellus* required for 10-fold in LC_50_ of triflumezopyrim (*h^2^* = 0.09) at different selection intensities.

After continuous TFM resistance selection for 21 generations, the average selection response (*R*) was 0.07, the average selection differential (*S*) was 0.73, and the *h* ^*2*^ was 0.09 (Table 2). As the selection pressure increased in each generation (survival rate after selection was from 20% to 90%), the generation that showed a 10-fold increase in resistance to TFM decreased from 40 to 8 generations (Figure 2).

**TABLE 2:** Realized heritability of SBPH to TFM.

Mean selection response per generation				Mean selection differential per generation				Realistic heritability (*h* ^*2*^)
Initial LC_50_ (mg·L^−1^)	Final LC_50_ (mg·L^−1^)	Selective response (*R*)	Survival rate (*p*)	Select intensity (*i*)	Average slope	Phenotypic standard deviation (*σ*_*p*_)	Selection differential (*S*)	0.09
0.55	14.46	0.07	40.1	0.97	1.33	0.75	0.73	

### 3.3 Resistance stability of the RS strain

To investigate the resistance stability of TFM, the RS strain (F_16_) of SBPH was continuously reared for 5 generations without TFM exposure. The RS strain resistance decreased from 13.05-fold (F_16_) to 11.00-fold (RD_5_), and the average selection response (*R*) was -0.01 (Table 3). However, there is no statistical decline in resistance from F_16_ to RD_1-5_.

**TABLE 3:** The resistance stability of RS strain of SBPH without TFM.

Generations	No.	Slope±SE	LC_50_ mg·L^−1^	95% CI (mg·L^−1^)	*χ* ^2^ (*df*)	Resistance ratio (RR)	The average selection response (*R*)	LCR_50_ ^a^ (95% CI)^c^	Hypothesis of equality (*χ* ^*2*^, *p*)^d^
F_16_	360	1.26 ± 0.32	7.18	4.31–11.32	2.88 (5)	13.05	−0.01		
RD_1_ ^b^	360	1.64 ± 0.28	7.35	5.24–10.08	2.08 (5)	13.36		0.98 (0.57–1.68)	1.85.0.40
RD_2_	360	1.34 ± 0.27	6.99	4.56–10.21	7.29 (5)	12.71		1.03 (0.58–1.83)	0.34.0.84
RD_3_	360	1.44 ± 0.27	7.04	4.76–10.02	6.17 (5)	12.80		1.02 (0.58–1.79)	0.74.0.69
RD_4_	360	1.13 ± 0.26	6.65	3.90–10.34	5.19 (5)	12.09		1.08 (0.58–2.01)	0.06.0.97
RD_5_	360	1.28 ± 0.27	6.05	3.69–8.89	4.14 (5)	11.00		1.19 (0.66–2.15)	0.60.0.74

^a^ LCR_50_ means LC_50_ of F_16_/LC_50_ of RDn.

^b^ RD, means Resistance decline strain.

^c^ The difference is significant if the 95% confidence limit is greater than 1 and the *p* values for the equality hypothesis is less than 0.05.

### 3.4 Cross-resistance of the RS strain

The results of cross-resistance between TFM and other insecticides for the RS strain of SBPH are shown in Table 4. Compared to SS strain, the cross-resistance ratio of the RS strain for imidacloprid, nitenpyram, thiamethoxam, dinotefuran, flonicamid, pymetrozine, and cyantraniliprole were 1.48, 1.28, 1.10, 0.90, 0.95, 1.39, and 1.08-fold, respectively. All cross-resistance ratio were lower than 2. The TFM had no cross-resistance with these insecticides.

**TABLE 4:** Cross-resistance of SS and RS strains of SBPH to seven insecticides.

Insecticides	Strains	No.	Slope±SE	LC_50_ (95%CI) (mg·L^−1^)	*χ* ^2^ (*df*)	Cross-resistance ratio (CR)
Imidacloprid	SS	360	1.21 ± 0.21	18.79 (11.65–27.34)	10.57 (6)	-
	RS	360	1.16 ± 0.20	27.73 (18.32–41.74)	7.57 (6)	1.48
Nitenpyram	SS	360	1.27 ± 0.32	1.90 (1.09–3.22)	1.78 (5)	-
	RS	360	1.38 ± 0.33	2.44 (1.56–4.29)	2.57 (5)	1.28
Thiamethoxam	SS	360	1.35 ± 0.33	5.37 (2.71–8.41)	3.24 (5)	-
	RS	360	1.34 ± 0.33	5.92 (3.13–9.34)	2.95 (5)	1.10
Dinotefuran	SS	360	1.45 ± 0.27	3.89 (2.68–5.59)	4.71 (5)	-
	RS	360	1.33 ± 0.27	3.51 (2.29–5.13)	6.25 (5)	0.90
Flonicamid	SS	360	1.20 ± 0.36	8.16 (3.86–13.76)	1.73 (5)	-
	RS	360	1.54 ± 0.38	7.76 (4.46–11.54)	2.93 (5)	0.95
Pymetrozine	SS	360	1.26 ± 0.32	28.40 (16.15–48.16)	1.61 (5)	-
	RS	360	1.577 ± 0.34	39.60 (26.73–66.29)	2.06 (5)	1.39
Cyantraniliprole	SS	360	1.17 ± 0.32	16.02 (7.86–27.34)	2.43 (5)	-
	RS	360	1.32 ± 0.33	17.25 (9.75–27.91)	2.23 (5)	1.08

### 3.5 Biological fitness comparison between the RS and susceptible strain strains

**FIGURE 3:**
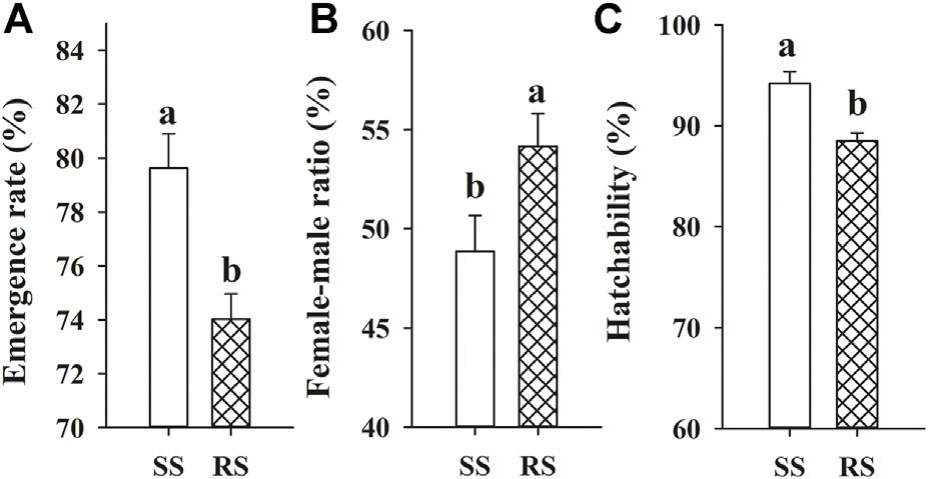
Means (±SE) of emergence rate **(A)**, female-male ratio **(B)** and hatchability **(C)** of SBPH in the SS and RS strains. Different letters above error bars are significantly different at 0.05 levels using the Tukey’s test.

The development duration and life table parameters of the SS and RS strains of SBPH are shown in Table 5; Figure 3, respectively. The development time of third instar, fifth instar and pre-adult, adult preoviposition period (APOP), longevity of the RS strain were significantly reduced by 0.39, 0.59, 0.99, 0.85, and 3.93 days, respectively (*p* < 0.05) as compared to SS strain (Table 5). Further, the emergence rate and hatchability were significantly decreased in the RS strain by 5.6% and 5.7%, respectively (*p* < 0.05). In contrast, the female-male ratio was substantially increased by 5.3% (*p* < 0.05) compared to that of the SS strain (Figure 3). No statistically substantial differences were observed in any other parameters (*p* > 0.05). Though no significant differences of *R*_*0*_ were found, *R*_*0*_ (47.52) of the RS strain was lower than the *R*_*0*_ (53.24) of the SS strain, and the relative fitness *R*_*f*_ was 0.89 (Table 5).

**TABLE 5:** Duration of the development and life table parameters for the SS and RS strains of SBPH^a^.

Stages		Susceptible strain (SS)	Resistance strain (RS)
Nymphal stage duration (d)	1st instar	2.89 ± 0.05^a^	2.78 ± 0.05^a^
	2nd instar	2.38 ± 0.06^a^	2.36 ± 0.05^a^
	3rd instar	2.70 ± 0.07^a^	2.31 ± 0.06^b^
	4th instar	3.26 ± 0.13^a^	3.22 ± 0.10^a^
	5th instar	4.78 ± 0.16^a^	4.19 ± 0.10^b^
	Pre-adult	15.87 ± 0.22^a^	14.88 ± 0.22^b^
Adult stage duration (d)		17.75 ± 0.84^a^	17.45 ± 0.81^a^
APOP^b^ (d)		5.44 ± 0.23^a^	4.59 ± 0.19^b^
TPOP^c^ (d)		19.96 ± 0.25^a^	20.38 ± 0.23^a^
Longevity(d)		29.76 ± 1.24^a^	25.83 ± 1.25^b^
Fecundity (eggs/female)		133.95 ± 9.11^a^	122.92 ± 7.32^a^
Emergence rate^d^ (%)		79.63 ± 1.27^a^	74.03 ± 0.93^b^
Female-male ratio^d^ (%)		48.87 ± 1.8^b^	54.17 ± 1.64^a^
Hatchability^d^ (%)		94.2 ± 1.17^a^	88.5 ± 0.80^b^
*r* (d^−1^)		0.15 ± 0.01^a^	0.15 ± 0.01^a^
*λ* (d^−1^)		1.17 ± 0.01^a^	1.16 ± 0.01^a^
*R*_*0* (offspring individual-1)_		53.24 ± 7.44^a^	47.52 ± 6.66^a^
*T* (d)		25.61 ± 0.47^a^	25.45 ± 0.44^a^
*R*_*f*_		-	0.89

^a^ The values (mean ± SE) followed by different letters in the same row indicate the significant difference at *p* < 0.05 using the paired bootstrap test.

^b^ APOP, means adult preoviposition period.

^c^ TPOP, means total preoviposition period.

^d^ The values (mean ± SE) followed by different letters in the same row are significantly different at 0.05 level using the Tukey’s test.

### 3.6 Synergism of the enzyme inhibitors

The synergism results of enzyme inhibitors showed that (Table 6), PBO exhibited significant synergistic action on the RS strain with synergistic ratios of 1.88-fold, but no significant synergistic action on the SS strain with synergistic ratios of 1.52-fold. TPP showed no significant synergistic action on the two strains, with a low synergistic ratio1.18- and 1.28-fold, respectively. DEM also had no synergism on two strains, only with synergistic ratios of 1.02- and 1.07-fold, respectively.

**TABLE 6:** Synergism of three enzyme inhibitors on TFM of SBPH.

Strain	Treatment	No.	Slope±SE	LC_50_ (mg·L^−1^) (95% CI)	*χ* ^2^ (*df*)	Synergistic ratios^a^ (SR) (95% CI)^b^	Hypothesis of equality (*χ* ^*2*^, *p*)^c^
Sensitive strain (SS)	TFM	360	1.61 ± 0.34	0.57 (0.38–0.90)	3.20 (5)	-	
	TFM + TPP	360	1.34 ± 0.27	0.49 (0.33–0.72)	4.96 (5)	1.18 (0.69–2.02)	0.78.0.68
	TFM + DEM	360	1.64 ± 0.28	0.56 (0.40–0.77)	6.47 (5)	1.02 (0.62–1.69)	0.01.0.99
	TFM + PBO	360	1.37 ± 0.27	0.38 (0.25–0.55)	5.05 (5)	1.52 (0.89–2.60)	2.7.0.26
Resistance strain (RS)	TFM	360	1.44 ± 0.27	8.25 (5.74–11.99)	4.71 (5)	-	
	TFM + TPP	360	1.13 ± 0.29	6.46 (4.07–10.64)	5.08 (5)	1.28 (0.73–2.24)	1.56.0.46
	TFM + DEM	360	1.24 ± 0.26	7.70 (5.17–12.70)	3.62 (5)	1.07 (0.63–1.84)	0.39.0.82
	TFM + PBO	360	1.36 ± 0.33	4.39 (2.73–7.45)	3.62 (5)	1.88^b^ (1.06–3.33)	4.59.0.04

^a^ SR (synergism ratio) = LC_50_ of TFM/LC_50_ of TFM + synergist.

^b^ The difference is significant if the 95% confidence limit is greater than 1 and the *p* values for the equality hypothesis is less than 0.05.

^c^ Significant synergistic action.

### 3.7 Detoxification metabolic enzyme activity

**FIGURE 4:**
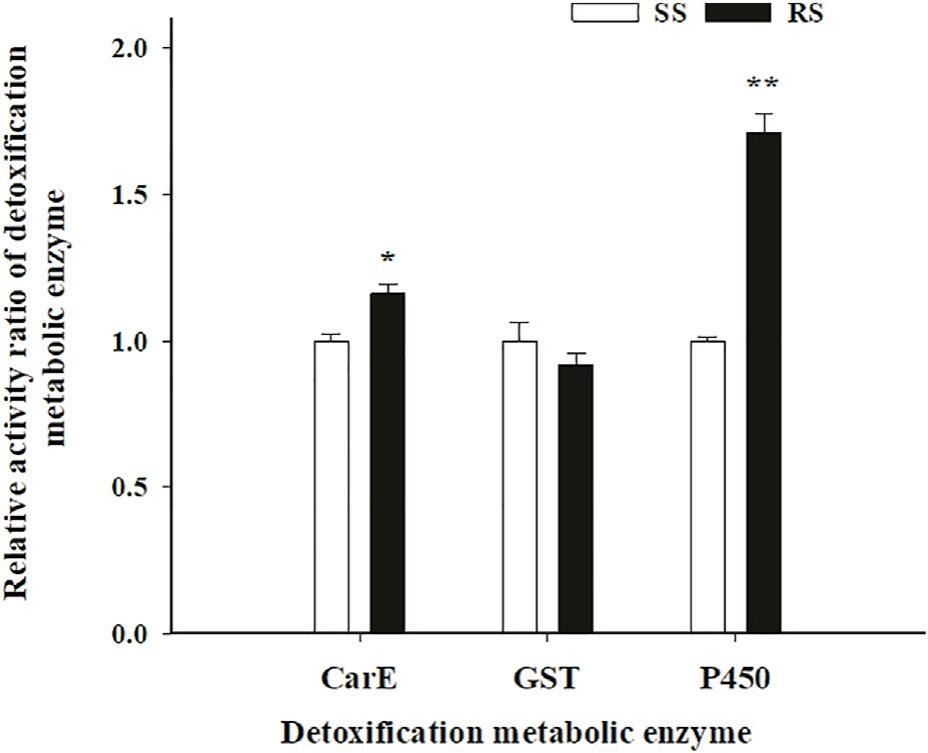
Means (±SE) of activity ratio of detoxification metabolic enzyme in SS and RS strains of SBPH. *Significant at 0.05 level and **Significant at 0.01 level using the Tukey’s test.

The activity of the three detoxification metabolic enzymes (CarE, GST, and P450) in the SS and RS strains of SBPH was shown in Figure 4. Compared to the SS strain, the activity of P450 in the RS strain increased significantly by 1.71-fold (*p* < 0.01), CarE in the RS strain also increased by 1.16-fold (*p* < 0.05). However, the GST activity showed no significant differences between in the SS and RS strains. According to the enzyme activity and synergism results, P450 and CarE may be both contribute to metabolic resistance of SBPH to TFM, and P450 may be one major metabolic factor.

### 3.8 Expression levels of P450 genes in RS and susceptible strain strains

**FIGURE 5:**
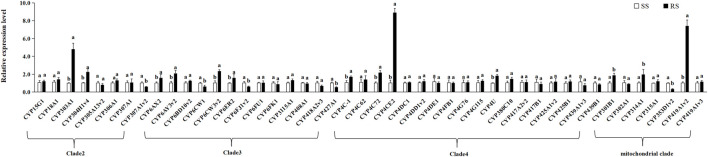
Means (±SE) of relative expression levels of P450 genes. Different letters above error bars for the same gene indicates significant differences between the RS and SS strains at 0.05 levels using the Tukey’s test.

To further clarify the association between P450 genes and the TFM resistance mechanism of SBPH, we determined and compared the differences in the expression levels of 53 P450 genes between the SS and RS strain. The up-regulated expression of nine genes (*CYP303A1*, *CYP304H1v4,* and *CYP305A13v2* from Clade 2; *CYP6CW3v2* and *CYP6ER2* from Clade 3; *CYP4C72*, *CYP4C78*, and *CYP4CE2* from Clade 4; *CYP419A1v2* from the mitochondrial clade) was more than 2-fold in the RS strain. In particular, the expression levels of *CYP303A1*, *CYP4CE2,* and *CYP419A1v2* were 4.82-, 8.92-, and 7.41- fold, respectively (Figure 5).

## 4 Discussion

SBPH has developed resistance to many insecticides due to the indiscriminate application of pesticides in the field (Elzaki et al., 2015; Liu et al., 2015). TFM is a novel insecticide and the only member of the 4E group to target nAChR (Cordova et al., 2016; Wen et al., 2021a). TFM is highly effective in controlling SBPH (Zhang Y. C et al., 2020). However, the resistance risk and underlying mechanism of SBPH against TFM have few studies reported yet.

After 21 generations of continuous selection with TFM, SBPH developed a 26.29-fold resistance to TFM. The resistance development showed a fluctuating upward trend. The SBPH slowly developed resistance against TFM till the F_8_ generation. But the resistance was rapidly increased to 26.29-fold after the F_8_ generation, reaching the middle resistance level. However, previous study showed SPBH could increase 45.1-fold to TFM only after 16 generations selection (Zhang et al., 2022). This different result may be due to the differences of selection method. Because the insecticide treated times were 96 h in previous study, which gave more selection pressure to SBPH than in our study, in which the treated times were only 72 h.

In the previous research, the resistance development trend of SBPH for buprofezin showed an “S” curve. During buprofezin resistance selection, the resistance increased slowly in the early stage and then increased exponentially after reaching a critical value. The resistance remained relatively stable after reaching a certain level (Zhang et al., 2012; Mao et al., 2016). The differences between our findings and those of other studies may be still due to insufficient time for resistance selection in our experiment. Therefore, the resistance selection of SBPH will need to be continued to obtain more realistic development trends and laws for resistance of SBPH to TFM.

Evaluating the resistance risk of new insecticides can provide critical theoretical references for scientific use and resistance prevention. The *h* ^*2*^ for resistance represents the ability of insect resistance to be inherited by the next generation and the risk of resistance development. Diptaningsari et al. (2019) reported that the *h* ^*2*^ of *Nilaparvata lugens* (Stål) for imidacloprid was 0.0893. Zhang Y. C et al. (2020) estimated that during the development of TFM resistance in *N. lugens*, *h* ^*2*^ was 0.0451. Recent studies have reported that the *h* ^*2*^ of TFM resistance in SBPH was 0.13 (Zhang et al., 2022). When the TFM selection pressure was used with a survival rate of 50% in each generation, the LC_50_ values could increase 10-fold only in 6.72 generations. However, we found that the *h* ^*2*^ of TFM resistance in SBPH was 0.09, which was lower than Zhang et al.‘s results. The resistance could increase 10-fold in just 18.00 generations when the survival rate was 50% for each generation. Therefore, if TFM is consistently used in the field, SBPH will develop a specific TFM resistance.

Fu et al. (2018) determined the resistance stability of the spinetoram-selected strain of Thrips hawaiiensis and found that without insecticide exposure, the resistance decreased from 19.42-fold to 12.35- and 9.50-fold after two and five generations, respectively, and the average reaction rates were -0.0982 and -0.0621, respectively. In this study, the resistance level decreased 2.05-fold after 5 generations without TFM, but there were no statistical decline in resistance level from F_16_ to RD_1-5_. The TFM resistance stability of SBPH was related high, which may be able to lead to problems for future resistance management of TFM.

TFM generally has no cross-resistance with other insecticides, including nAChR competitive regulators. Several studies have reported that TFM has no cross-resistance with sulfoxaflor, nitenpyram, clothianidin, and buprofezin (Mao et al., 2018; Liao et al., 2019a; Jin et al., 2019; Zeng et al., 2022). Our results showed that there was no evidence of cross-resistance between TFM and tested five neonicotinoids insecticides, pyridine azomethine insecticide and anthranilic diamide insecticide. However, in Zhang et al.’s study (2022), the triflumezopyrim-resistant strain of SBPH showed minor cross-resistance to dinotefuran, in which may be due to the resistant level of previous study (45.1-fold) were higher than our research (26.29-fold). These results indicate that TFM may have a certain risk of cross-resistance to neonicotinoids insecticides in future.

Determining fitness cost and the relationship between resistance fitness and resistance are significant for clarifying the law of resistance development. Several studies have shown that resistant insect strains display fitness costs under insecticide selection pressure. Jin et al. (2021) showed that the *R*_*f*_ of clothianidin-resistant strains of N. lugens was 0.78. Compared with the SS strain, the APOP of clothianidin-resistant strains increased significantly, while fecundity decreased significantly. Zhang et al. (2018) studied the difference in life table between nitenpyram-resistant *N. lugens* and its susceptible strain by an age-stage, two-sex life table method and found that the fitness cost of the resistant strain was 0.55. Moreover, the *r* and *R*_*0*_ values were lower than those of the susceptible strain. The developmental duration of the resistant strain was increased, whereas life span and hatchability were significantly decreased. In our study, the third instar, fifth instar, pre-adult, APOP, longevity, emergence rate, and hatchability of the RS strain were significantly reduced compared to the SS strain. The *R*_*f*_ value of the RS strain was 0.89. Therefore, the RS strain has disadvantages in development and reproduction. These adverse effects of fitness cost are important factors limiting resistance evolution, which may be valuable in formulating effective resistance management strategies (Kliot and Ghanim., 2012).

The metabolic resistance mechanism of SBPH to TFM can be revealed by comparing the activities of three detoxification metabolic enzymes and the synergism of enzyme inhibitors between the SS and RS strains. Several studies have demonstrated that increased activity of metabolic detoxification enzymes contributes significantly to insects’ resistance to insecticides (Zhang S. R et al., 2020). Previously, Lu et al. (2017) found that TPP can effectively reduce the resistance level of *N. lugens* to chlorpyrifos, which demonstrated that the resistance of *N. lugens* to chlorpyrifos was related to an increase in CarE activity. The resistance of *N. lugens* to nitenpyram has also been associated with increased CarE activity (Zhang et al., 2017). However, previous study found that CarE activity was not associated with TFM resistance in SBPH (Zhang et al., 2022). In this study, although CarE enzyme activity in resistant strain was higher than that in susceptible strain, TPP had no obvious synergistic effect on the two strains.

The increase in GST activity was related to the resistance of insect pests to pyrethroids, neonicotinoids, and other insecticides (Vontas et al., 2001; Yang et al., 2016; He et al., 2018). However, based on the results of enzyme inhibitor synergism and activity, GST did not play a role in the resistance development in SBPH for TFM, similar to Zhang et al results (2022).

P450 is an important enzyme in detoxification. Many studies have shown that P450 can mediate the resistance of insects to insecticides (Tabashnik. 1992; Wen et al., 2009; Ding et al., 2013; Garrood et al., 2016). Mao et al. (2016) studied the resistance mechanism of SBPH for buprofezin and found that the enhanced P450 activity plays an essential role in the resistance of SBPH to buprofezin. Zhang et al. (2022) also found that P450 contribute to triflumezopyrim resistance in *L. striatellus*. Similar to previous studies, both the increases of P450 activities and higher synergistic ratio of PBO were found in resistant strain. The enzyme activity and synergism results indicated that P450 and CarE may be both contribute to metabolic resistance of SBPH to TFM, and P450 may be one major metabolic factor. More studies will need to be performed to confirm if CarE contributed to TFM resistance in SBPH in future.

To further clarify the detoxification enzymes involved in developing TFM resistance in SBPH, we selected 53 P450 genes for their expression analysis based on the enzyme activity and enzyme inhibitor results. The overexpression of the P450 genes generally causes the enhancement of P450 activity in resistant insects (Elzaki et al., 2016). Previous studies reported overexpression of *CYP6AY3v2*, *CYP353D1v2*, and *CYP4C71* in resistant SBPH strains. These genes encode the P450 enzymes, which can metabolize imidacloprid and mediate the generation of resistance to imidacloprid (Elzaki et al., 2016; Elzaki et al., 2017; Wang et al., 2017; Xiao et al., 2019). Zhang et al. (2022) found that seven P450 genes were up-regulated more than 1.5-fold in TFM-resistance strain of SBPH, but only three genes were up-regulated more than 2-fold. In our study, compared with SS strain, nine genes were more than 2-fold overexpressed in the RS strain, which *CYP303A1*, *CYP4CE2*, and *CYP419A1v2* were significantly up-regulated.

Previous studies have shown that the resistance of *Bemisia tabaci* field population to imidacloprid may be related to the increased expression of *CYP303A1-like* gene. (Wang Q. et al., 2020). The significant overexpression of *CYP303* gene has a significant correlation with the formation of resistance of *Bemisia tabaci* to imidacloprid in the field. (Ilias et al., 2015). Therefore, *CYP303A1* may play a role in the resistance of SBPH to TFM. However, Zhang et al. (2012) found that the expression levels of *CYP303A1* and *CYP419A1v2* genes were not related to the resistance of *L. striatellus* to thiamethoxam. Also, there were no significant differences in *CYP4CE2* expression between deltamethrin-resistant population and sensitive population of *L. striatellus* (Xu et al., 2013). Next, further studies are still needed to prove whether these three genes are associated with TFM resistance in *L. striatellus*. In summary, the results of this study on the resistance risk and metabolic resistance mechanism of SBPH for TFM could assist in the rational application and prolong the insecticide service life in the field context. Moreover, these findings will provide an essential theoretical reference for delaying the development of resistance to TFM and resistance management.

## Data Availability

The original contributions presented in the study are included in the article/Supplementary Material, further inquiries can be directed to the corresponding author.
