# Supplementary material for: Resistance selection of triflumezopyrim in Laodelphax striatellus (fallén): Resistance risk, cross-resistance and metabolic mechanism
Source: Front Physiol. 2022 Nov 29;13:1048208. doi: 10.3389/fphys.2022.1048208 (PMC9745130; doi:10.3389/fphys.2022.1048208)
Supplement: Supplementary file 1 [file Presentation1.zip › Supplementary Table.docx]

**Table S1.** Primer pairs used for qRT-PCR relative quantification of gene expression in SBPH.

|  | Gene name | Forward primer | Reverse primer |
| --- | --- | --- | --- |
| CYP2  Clade | *CYP15G1* | ACAAGCAAATGTGGGAAAG | ATCGGTGTGAGAGAAGCG |
|  | *CYP18A1* | TTGGAAAGTGTCGGAAAAC | CGAAAATGAAGATGGGTGT |
|  | *CYP303A1* | GTTTCGTTTTGCGTCACCT | CTGCTTTTTCTCACCCTCG |
|  | *CYP304H1v4* | TTAGCGAGTCTCCGAAAC | CATCCACATACATCCTCAAGT |
|  | *CYP305A13v2* | GTTCTACTTGCTCTTCTGCT | CCTCCCAACTTTCTACTCA |
|  | *CYP306A1* | TTTTTAGGGGCTACGGTTTA | CTCGTTTCGGATGCGATTTA |
|  | *CYP307A1* | GAAACGGATATCGGGATG | GGCTGCTTATGGTAATGGA |
|  | *CYP307A1v2* | AGCAGCTGAATATTGGGA | GGATGAAGTACTCGGGTTT |
| CYP3  Clade | *CYP6AX2* | GTGGGGGACAGAGTGGTAT | CGCTTGTTTTCTTCAGTGAA |
|  | *CYP6AY3v2* | ATTGGGTCTCTCTGAATTTC | TTGAGTCCATGCCTAGGT |
|  | *CYP6BD10v2* | CATTATTCAGTTGTCCCACC | TCATGTACCACACCTCTCTTC |
|  | *CYP6CW1* | TTGGAGAATGTGCTGAAC | CCTGTGAATGCCTATGAC |
|  | *CYP6CW3v2* | CGCTTTTTCAGTTCTTCCA | TTCTGCGTCATTCAGTTTC |
|  | *CYP6ER2* | GACCTTCCTCTTCATCTTG | TTGCCATCCATTGTATCG |
|  | *CYP6FJ1v2* | AGGAAGCAGACAAAGAGG | GAACGAATGTGCAATCAC |
|  | *CYP6FU1* | TATTTGTTTCCCGCTCTC | AGTTGTCCTTGGTCACTTTC |
|  | *CYP6FK1* | GGGTCCAAGGAACTGCATAG | GAGAGTTTCAGCCAAATGCC |
|  | *CYP3115A1* | GGCCTTGACTTTGACTCG | CACTTTGGGCATACCGAT |
|  | *CYP408A1* | ATATCGGCGTTCAACTTG | CTTGTTCTTCTGCGGTAG |
|  | *CYP418A2v3* | TTGGCTTTTTCAAATCCACTCC | TTCGACGATCCAGACCTCG |
|  | *CYP427A1* | ATTCGGCAAGTCCCATAG | CCAGTAACCAAACAACCAT |
| CYP4  Clade | *CYP4C-1* | TCTCCTTCAATGCAAGACC | CCGTTAAGCCGACGACTA |
|  | *CYP4C62* | TCAGACTATTTCCCAGCG | GGTTCATAACACATCCAGC |
|  | *CYP4C72* | TTGGCACTGGACTTCTTAC | GAATGTCGCTCTGCTCTC |
|  | *CYP4CE2* | GCGAGCAATATGTGAGAG | GTGTGAAGGAGTGTAGAAC |
|  | *CYP4DC1* | GACACTCTGCTGAACTAC | CAGAAGATGTTGTTGTATCG |
|  | *CYP4DD1v2* | GAGATTGAGGAGGTATTTGG | TCTTCTGTGACAGTGCGAG |
|  | *CYP4DE1* | GCAGGTCAAAACACAACTC | TCTCCAACTCCTCAAACG |
|  | *CYP4FB1* | TGCTCTTCTTATGCTGGC | TTCCTTGATGACTCGTTCC |
|  | *CYP4G76* | TTTCCACCTGTTCCCCTC | CCACCACTGTTGTATTCTTTG |
|  | *CYP4G115* | AGGAAGAAGTTGACACCAT | TCTGAGTTGCCGAATACC |
|  | *CYP4U* | CGCTCACTGGCGTTTAGT | GCGGAGGACGTTTTTTAA |
|  | *CYP380C10* | GCATACCGACTTTGTGTTC | CTTTCCTGCTCTTTCTTCTT |
|  | *CYP417A2v2* | CGAAATACAGCTATCTACCG | TTATGACACTTCCCACCA |
|  | *CYP417B1* | AGACTACACTACCTCGAAATGG | AACTGCGTAAAGACTCAACAG |
|  | *CYP425A1v2* | AACTTCTGGAGAGGTGTGGA | GTTTCTTGGGGAGATTTTTG |
|  | *CYP425B1* | GGAAACATCAAAGGGTCTG | TGGCGTAATCTAGCATCAC |
|  | *CYP439A1v3* | CGAAGACATACAAGAAAAGG | TGTGAGGAGGACAGCAGT |
|  | *CYP439B1* | TCAACAATACACTTCTTGGC | GTAGGGGCTCTTTACTTCC |
| mitochondrial  clade | *CYP301B1* | GAAAGACATAGAAGCCAAGG | CGACAAGAAACATATCCAGAG |
|  | *CYP302A1* | GCTCAGAAAGCCACCTACA | TCCGCTCATGACAACATC |
|  | *CYP314A1* | GGCGTTAGTAGTTGTATTCC | CGGCTTCTCCTCTATTATCA |
|  | *CYP315A1* | ACGGAATGGGATTGAGAAG | CGCAGGTTGAAACAAGATG |
|  | *CYP353D1v2* | TATGGAGGTGTTTCTTGGT | TGAGCTTCTGGATTCTGAG |
|  | *CYP419A1v2* | GAAGCATCCTATACCATACTC | TACCTCATTGGCTGTCAT |
|  | *CYP419A1v3* | TGTGGAGGGTGTATGAAA | CAGGTTGGATTGTAGCATT |
| Reference gene | *GAPDH* | CTACTCATCCCGTGTCATCG | ATCAAAGTCGAAGGCCTGAT |
