# Supplementary material for: Resistance selection of triflumezopyrim in Laodelphax striatellus (fallén): Resistance risk, cross-resistance and metabolic mechanism
Source: Front Physiol. 2022 Nov 29;13:1048208. doi: 10.3389/fphys.2022.1048208 (PMC9745130; doi:10.3389/fphys.2022.1048208)
Supplement: Supplementary file 1 [file Presentation1.zip › Supplementary.docx]

**S1. Calculation of resistance realistic heritability and resistance development rate**

The realistic heritability (*h^2^*) for TFM resistance was estimated using the method proposed by Tabashnik and Mcganghey^20^. The formula is as follows:

$$\text{h}^{\text{2}}\text{=}\frac{\text{R}}{\text{S}}$$

where *R* is the selection response calculated as follows:

$$\text{R}\text{=}\frac{\lg(\text{final }\text{LC}_{\text{50}}\text{)}\text{-l}\text{g(initial }\text{LC}_{\text{50}}\text{)}}{\text{n}}$$

while *S* is the response of selection differential, which was calculated as follows:

$$\text{S}\text{=}\text{i}\text{×}\text{σ}_{\text{p}}$$

where *i* is the select intensity. The formula is as follows:

$$\text{i}\text{≈1.583-0.0193336P+0.0000428}\text{P}^{\text{2}}\text{+}\text{3.65194/P}$$

where P is the response of the average survival rate (%) of each generation in the resistance selection.

*σ_p_* is the phenotypic standard deviation and is calculated as follows:

$$\text{σ}_{\text{p}}\text{=}\left( \frac{\text{1}}{\text{n}}\sum_{\text{i=1}}^{\text{n}} \text{b}_{\text{i}} \right)^{\text{-1}}$$

where *b_i_* refers to the slope of each generation in resistance selection.

The rate of resistance development was calculated according to the method described by Tabashnik^21^. The formula for predicting the required number of generations for a 10-fold increase under different selection pressures is as follows:

$$\text{G}_{\text{10}}\text{=}\frac{\text{1}}{\text{h}^{\text{2}}\text{s}}$$

**S2. Determination steps of detoxification enzyme activity**

Thirty 3^rd^ instar nymphs were homogenized in 1 mL of sodium phosphate buffer (0.1 mol·L^-1^ (pH 7.6) containing 1 mmol·L^-1^ ethylene diamine tetra acetic acid, 1 mmol·L^-1^ phenyl methyl sulfonyl fluoride, 1 mmol·L^-1^ phenyl thiourea, and 20% glycerol) on ice. The crude homogenates were centrifuged at 10,000 rpm for 15 min at 4 °C, and the supernatant was used as the enzyme source to measure the detoxification-enzyme activity. Three replicates were used for each population (90 insects per population).

CarE activity was evaluated according to the methods reported by Han et al.^24^ and Ding et al.^25^, with minor modifications. Solid Blue RR salt (20 mg) and 0.2 mL of 100 mmol·L^-1^ α-naphthyl acetate ethanol solution were added to 10 mL of 0.2 mol·L^-1^ phosphoric acid buffer at pH 6.0. The mixture was shaken, mixed, and filtered to obtain the substrate and developer. A 20-μL aliquot of diluted enzyme solution (diluted 10 times with 0.1 mol·L^-1^, pH 7.6 phosphate buffer in advance) and 205 μL of the mixed solution of substrate and developer were added to each well of a 96-well standard plate. The reactions were monitored every 30 s for 15 min at a wavelength of 450 nm and at 27 ºC using an Epoch2 multifunction microplate reader.

Glutathione-S-transferase (GST) activity was analyzed using the method described by Kao et al.^26^, with slight revisions. Briefly, 50 μL of 0.6 mmol·L^-1^ 1-chloro-2,4-dinitrobenzene (CDNB) and 100 μL of 6 mmol·L^-1^ reduced glutathione (GSH) were added to an ELISA plate, followed by the addition of 100 μL of enzyme solution. Changes in absorbance within 10 min were recorded every 20 s at a wavelength of 340 nm and at 27 °C using an Epoch2 multifunction microplate reader.

Cytochrome P450 monooxygenase (P450) activity was determined using the method described by Aitio et al.^27^, with some modifications. Briefly, 120 µL enzyme solution was mixed with 365 µL phosphate buffer (0.1 mol·L^-1^ pH 7.5), 5 µL 7-ethoxycoumarin (7-EC) (40 mmol·L^-1^), and 10 µL nicotinamide adenine dinucleotide phosphate (NADPH) (10 mmol·L^-1^). The test tubes were shaken at 30 °C for 15 min and then immediately placed on ice. A stop solution (300 µL) containing 15% trichloroacetic acid was added to the samples to terminate the reaction. The mixture was centrifuged at 15,000 × *g* for 2 min at 4 °C. Thereafter, 200 µL glycine-NaOH buffer (1.6 mol·L^-1^, pH 10.5) was added to 400 µL of the supernatant. Changes in absorbance within 10 min were recorded every 20 s at an excitation wavelength of 358 nm and emission wavelength of 465 nm using a Spark 10M multimode microplate reader at 27 °C.

The Enhanced BCA Protein Assay Kit (Beyotime Biotechnology) was used to generate a standard curve. A 20-μL aliquot of supernatant was added to an ELISA plate and then mixed with 200 μL of BCA liquid. The reaction mixture was incubated at 37 °C for 25 min, and then the absorbance was measured at a wavelength of 562 nm using an Epoch2 multifunction microplate reader. The protein concentration of each sample was calculated.
